# Chronic pain intensity predicts long-term depressive disorders in older community-dwellers

**DOI:** 10.1192/j.eurpsy.2026.11481

**Published:** 2026-07-31

**Authors:** I. Rouch, M. Koleck, A. Edjolo, B. Laurent, H. Amieva

**Affiliations:** 1 neurology, Saint Etienne hospital, Saint Etienne; 2 U1219, INSERM, Bordeaux; 3 Saint Etienne hospital, Saint Etienne; 4 U1028, INSERM, Lyon, France

## Abstract

**Introduction:**

Chronic pain (CP) in older adults was associated with depression in numerous cross-sectional studies. However, the longitudinal link between CP characteristics and depressive symptoms in this population remains unsettled.

**Objectives:**

We aimed to assess the prospective link between CP intensity and long-term depressive symptoms in a population-based cohort of older participants, considering numerous potential confounders.

**Methods:**

The study sample was gathered from the PAQUID study, a cohort study of older community-dwellers above 65. Information regarding CP characteristics was collected at the 3-year follow-up visit. The sample included the 713 participants who fulfilled this CP questionnaire. Three groups of CP were distinguished: no, mild and intense CP. Depressive symptoms were assessed with the Center for Epidemiologic Studies Depression scale (CES-D) scale every 2 to 3 years during 12 years. The association between CP and the 12-year CES-D course was investigated using a multivariate latent process mixed model for curvilinear data.

**Results:**

Compared with the participants without CP, those with moderate and intense CP had significantly higher CES-D levels at baseline with a dose-response relationship as a function of pain intensity (β =0.391, p=0.0224, and 0.696, p <. 0001, respectively). However, the time course was comparable in the three groups (β =-0.003, p=0.8406, and 0.001, p = 0.9617, for mild and intense CP respectively).

**Conclusions:**

CP intensity is associated with higher levels of depressive symptoms persisting throughout the long period of follow-up. This finding reinforces the importance of early treatment of CP in older adults, to prevent its sustainable psychological consequences.

**Disclosure of Interest:**

None Declared

